# 1178. The Dalba Diary: A Retrospective Review of Dalbavancin Use, Clinical Outcomes, and Treatment Adherence Challenges at an Academic Medical Center

**DOI:** 10.1093/ofid/ofad500.1018

**Published:** 2023-11-27

**Authors:** Cassandra E Black, Eric Gregory, Nicole Wilson, D Matthew Shoemaker, Gabriel Ann Haas, Kellie Wark, Joanna Kimball

**Affiliations:** The University of Kansas Medical Center, Westminster, Maryland; The University of Kansas Health System, Kansas City, MO; The University of Kansas Medical Center, Westminster, Maryland; The University of Kansas Medical Center, Westminster, Maryland; Kansas Department of Health and Environment, Topeka, Kansas; University of Kansas, Kansas City, Kansas; The University of Kansas Medical Center, Westminster, Maryland

## Abstract

**Background:**

Dalbavancin is FDA-approved for skin and skin tissue infections (SSTI) and is an alternative therapy for deep-seated gram-positive infections when avoidance of long-term catheters or prolonged hospitalization for intravenous (IV) antibiotics is desired. This observational, retrospective study evaluated the utilization of dalbavancin at a large academic medical center between May 1, 2021, and November 30, 2021. The objectives were to analyze the patient characteristics, clinical outcomes (including treatment adherence), and economic impact by looking at the estimated length of stay (LOS) and cost avoidance associated with dalbavancin use.

**Methods:**

Variables of interest were analyzed with descriptive statistics. A chi-square test was used to assess the relationship between no-shows and infection-related readmissions. A multivariate logistic regression analysis was used to predict factors associated with treatment nonadherence.

**Results:**

We examined 96 cases consisting of 34 patients with IV drug use (35.4%), 47 with non-IV substance use (49.0%), and 22 with homelessness (24.0%). The most common indication for use was non-vertebral osteomyelitis (34.4%, *n*=33), followed by SSTI (16.7%, *n*=16) and endocarditis (14.6%, *n*=14) (Table 1). Treatment adherence was found to be 84.4% (*n*=81), and readmissions and adverse reactions were rare (Table 2). The average LOS avoided was 28 days (SD, 13.6). The average cost avoided was $56,000 US dollars (SD, $27,180.63). No-shows were not associated with infection-related readmissions (*p* =.99). In our multivariate analysis, those with non-IV substance use were significantly less likely to attend their scheduled infusion (*p*=.003), and those with higher Charlson Comorbidity Index (CCI) were significantly more likely to attend their infusion (*p*=.013) (Table 3).

**Figure:**
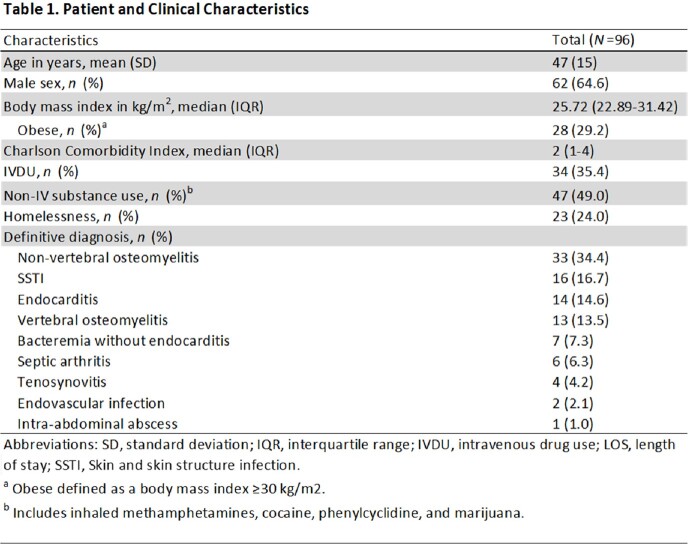

**Figure:**
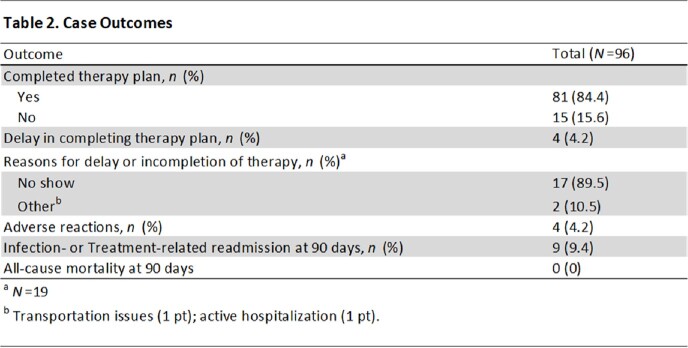

**Figure:**
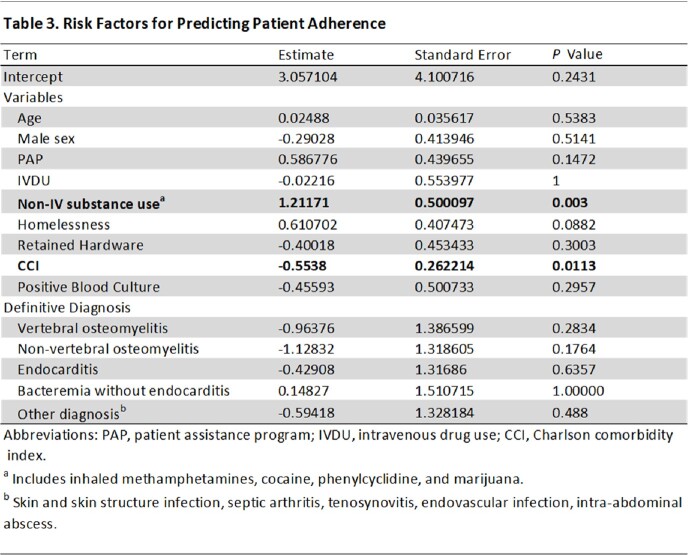

**Conclusion:**

Dalbavancin was a safe and economically beneficial alternative for deep-seated infections. Readmissions and adverse reactions were uncommon, and there were no documented deaths within 90 days of discharge. Treatment adherence was high. Those with non-IV substance use and possibly homelessness may be at higher risk for nonadherence, but treatment nonadherence did not lead to poorer outcomes.

**Disclosures:**

**All Authors**: No reported disclosures

